# Phosphate-modif ied CpG oligonucleotides induce in vitro maturation of human myeloid dendritic cells

**DOI:** 10.18699/VJ20.659

**Published:** 2020-10

**Authors:** A.A. Ostanin, O.Y. Leplina, E.A. Burakova, T.V. Tyrinova, A.A. Fokina, A.S. Proskurina, S.S. Bogachev, D.A. Stetsenko, E.R. Chernykh

**Affiliations:** Research Institute of Fundamental and Clinical Immunology, Novosibirsk, Russia; Research Institute of Fundamental and Clinical Immunology, Novosibirsk, Russia; Novosibirsk State University, Novosibirsk, Russia Institute of Cytology and Genetics of Siberian Branch of the Russian Academy of Sciences, Novosibirsk, Russia; Research Institute of Fundamental and Clinical Immunology, Novosibirsk, Russia Novosibirsk State University, Novosibirsk, Russia; Novosibirsk State University, Novosibirsk, Russia Institute of Cytology and Genetics of Siberian Branch of the Russian Academy of Sciences, Novosibirsk, Russia; Institute of Cytology and Genetics of Siberian Branch of the Russian Academy of Sciences, Novosibirsk, Russia; Institute of Cytology and Genetics of Siberian Branch of the Russian Academy of Sciences, Novosibirsk, Russia; Novosibirsk State University, Novosibirsk, Russia Institute of Cytology and Genetics of Siberian Branch of the Russian Academy of Sciences, Novosibirsk, Russia; Research Institute of Fundamental and Clinical Immunology, Novosibirsk, Russia

**Keywords:** monocytes, dendritic cells, differentiation, maturation, PAMP- and DAMP-activators, allo-MLR, CpG-oligonucleotide, моноциты, дендритные клетки, дифференцировка, созревание, PAMP- и DAMP-активаторы, алло-СКЛ, CpG-олигонуклеотид

## Abstract

Myeloid dendritic cells (DCs) play an important role in the immune response; therefore, the search for compounds that can effectively activate DCs is a needful goal. This study was aimed to investigate the effect of synthetic CpG oligodeoxynucleotides (CpG-ODN) on the maturation and allostimulatory activity of myeloid DCs in comparison with other PAMP and DAMP molecules. For the research, we synthesized known CpG-ODN class C (SD-101 and D-SL03) containing thiophosphate internucleotide groups, and their original phosphate-modified analogues (SD-101M and D- SL03M) with mesylphosphoramide internucleotide groups (M = μ-modification). The effects of CpG-ODN and other activators were evaluated on DCs generated from blood monocytes in the presence of GM-CSF and IFN-α (IFN-DC) or IL-4 (IL4-DC). Evaluation of the intracellular TLR-9 expression showed that both types of DCs (IFN-DC and IL4-DC) contained on average 52 and 80 % of TLR-9-positive cells, respectively. The CpG-ODNs studied enhanced the allostimulatory activity of IFN-DCs, and the effect of μ-modified CpG-ODNs was higher than that of CpG-ODNs with thiophosphate groups. The stimulating effect of CpG-ODN at a dose of 1.0 μg/ml was comparable (for D-SL03, D-SL03M, SD-101) with or exceeded (for SD-101M) the effect of LPS at a dose of 10 μg/ml. At the same time, IFN-DCs were characterized by greater sensitivity to the action of CpG-ODNs than IL4-DCs. The enhancement of DC allostimulatory activity in the presence of CpG-ODNs was associated with the induction of final DC maturation, which was confirmed by a significant decrease in the number of CD14+DC, an increase in mature CD83+DC and a trend towards an increase in CD86+DC. Interestingly, the characteristic ability of LPS to enhance the expression of the co-stimulatory molecule OX40L on DCs was revealed only for the μ-analogue SD-101M. In addition, CpG-ODNs (SD-101 and SD-101M) had a stimulatory effect on IFN-γ production comparable to the action of LPS. The data obtained indicate a stimulating effect of CpG-ODN on the maturation and allostimulatory activity of human myeloid DCs, which is more pronounced for μ-modified analogs.

## Abstract (ru)

Миелоидные дендритные клетки (ДК) играют важную роль в иммунном ответе, поэтому актуальной задачей является поиск соединений, способных эффективно активировать ДК. Целью настоящей работы было изучение влияния синтетических CpG олигодезоксинуклеотидов (CpG-ODN) на созревание и аллостимулятор- ную активность миелоидных ДК в сравнении с другими PAMP и DAMP молекулами. Для исследований были син- тезированы CpG-ODN класса С (SD-101 и D-SL03), содержащие тиофосфатные межнуклеотидные группы, а также получены их оригинальные фосфат-модифицированные аналоги (SD-101М и D-SL03М) с мезилфосфорамидны- ми межнуклеотидными группами (М = μ-модификация). Эффекты CpG-ODN и других активаторов оценивали в культурах ДК, генерированных из моноцитов крови в присутствии GM-CSF и IFN-α (IFN-ДК) или IL-4 (IL4-ДК). Оценка внутриклеточной экспрессии TLR-9 показала, что оба типа ДК (IFN-ДК и IL4-ДК) содержали в среднем 52 и 80 % TLR-9-позитивных клеток соответственно. Исследуемые CpG-ODN усиливали аллостимуляторную активность IFN-ДК, причем эффект μ-модифицированных CpG-ODN был выше, чем тиофосфатных CpG-ODN. Стимулирующий эффект CpG-ODN в дозе 1.0 мкг/мл был сопоставим (для D-SL03, D-SL03М, SD-101) или пре- вышал (для SD-101М) действие липополисахарида (LPS) в дозе 10 мкг/мл. При этом IFN-ДК характеризовались большей чувствительностью к действию CpG-ODN, чем IL4-ДК. Усиление аллостимуляторной активности ДК в присутствии CpG-ODN было связано с индукцией конечного созревания клеток, что подтверждалось значимым снижением количества СD14+ ДК, увеличением доли зрелых CD83+ ДК и тенденцией к возрастанию CD86+ ДК. Интересно, что характерная для LPS способность усиливать экспрессию костимуляторной молекулы OX40L на ДК была выявлена только для μ-аналога SD-101М. Кроме того, CpG-ODN (SD-101 и SD-101М) оказывали стиму- лирующий эффект на продукцию IFN-γ, сопоставимый с действием LPS. Полученные в целом данные свидетель- ствуют о стимулирующем действии CpG-ODN на созревание и аллостимуляторную активность миелоидных ДК человека, которое более выражено для μ-модифицированных аналогов.

## Introduction

Dendritic cells (DC) play an important role in immune responses, thus justifying their application as cellular targets and potential cellular modality for developing novel anti-cancer immunotherapies. Taking into account that only mature DC with high antigen-presenting and co-stimulatory molecule expression possess immunostimulatory activity (Banchereau et al., 2000), R&D efforts toward discovery of novel molecular candidates capable of effectively activating DC and induce their maturation are clearly very topical.

Pathogen-associated molecular patterns (PAMP) and damage- associated molecular patterns (DAMP) released upon autologous cell damage belong to natural DC activators. PAMP-dependent effects are mediated via pattern-recognition receptors, while DAMP molecules are recognised by intracellular sensors and activate DC via secondary messengers, such as tumour-necrosis factor α (TNF-α) (Jounai et al., 2013; Kawasaki, Kawai, 2014). The effects of various compounds on human DC are usually assessed in vitro in monocyte-derived DC cell cultures generated in the presence of GM-CSF/IL4 or GM-CSF/IFN-α (Cehim, Chies, 2019) cytokine combinations. In these settings, a Toll-like receptor 4 (TLR-4)-specific ligand, bacterial lipopolysaccharide (LPS), serves as a standard cell activator. However, LPS-associated pyrogenicity effectively prevents its clinical application.

Bacterial and viral DNA species are also capable of activating terminal DC maturation. This activity is accounted for by the presence of unmethylated CpG-dinucleotides in their structure, which could be imitated by synthetic CpG oligodeoxynucleotides (CpG-ODN) that mediate downstream signalling via TLR-9 (Polovinkina, Markov, 2010). Synthetic CpG-ODN derivatives have demonstrated pronounced immunostimulatory and anti-cancer effects in vivo, and therefore these compounds are currently considered as perspective adjuvants in anti-cancer immunotherapy (Scheiermann, Klinman, 2014; Shirota, Klinman, 2014; Shirota et al., 2015). CpGODNs along with plasmacytoid DC have been shown to exert stimulatory effects on bone marrow-derived DC in murine experimental systems (Behboudi et al., 2000). Meanwhile, CpG-ODN-dependent sensitivity in humans is attributed mainly to plasmacytoid DC, while CpG-ODN-mediated effects on myeloid DC were addressed in just a few studies that produced rather contradictory results (Behboudi et al., 2000; Hoene et al., 2006).

This study aimed to assess the effects of CpG-ODN on maturation and allostimulatory activity of myeloid DC generated from blood monocytes in the presence of GM-CSF and IFN-α (IFN-DC) or IL-4 (IL4-DC) in comparison with other PAMP (LPS) and DAMP activators. Specifically, we assessed the effects of CpG-ODN class С derivatives SD-101 (Levy et al., 2016) and D-SL03 (Yang et al., 2013) with thiophosphate internucleotide groups, as well as original phosphate-modified analogues (SD-101М, D-SL03М) with mesyl-phosphoramidate internucleotide groups (M = μ-modification) (Chelobanov et al., 2017).

In addition, we analysed the effects of DAMP activators: double-stranded DNA (dsDNA) and a synthetic polycationic adjuvant azoximer bromide (AB) (Kabanov, 2004; Powell et al., 2015), which was shown to enhance antigen-presenting DC function via activating pro-inflammatory signalling pathways (Dyakonova et al., 2004).

## Materials and methods

In this study the following compounds were synthesised, purified and characterised: CpG-ODN class С: SD-101 and D-SL03 containing thiophosphate (phosphorothioate) internucleotide groups, and original modified analogues (SD-101М, D-SL03М) with mesyl-phosphoramidate (μ) internucleotide linkages. CpG-ODN sequences used in this study are shown in Table 1.

**Table 1.**
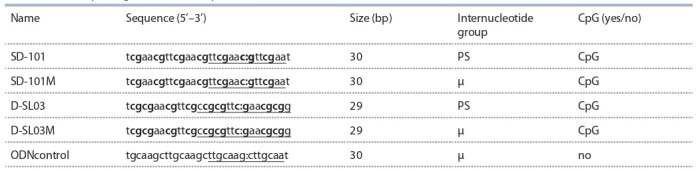
List of CpG-oligonucleotides (CpG-ODN) Notе. CpG dinucleotides are shown in bold; palindromic sequences are underlined; a middle of the palindromic sequence is indicated with the colon. PS – thiophosphate group; μ – mesylphosphoramide group.

DC were obtained from peripheral blood mononuclear cells (PBMC) isolated from heparinised venous blood of healthy donors using Ficoll-Verografin gradient centrifugation. IFN-DC were generated by cultivating an adherent PBMC fraction in Falcon (BD Biosciences, UK) flasks in RPMI-1640 medium (Sigma-Aldrich, St. Louis, MO, USA) supplemented with 0.3 mg/ml L-glutamine, 5 mM HEPES-buffer, 100 μg/ ml gentamycin and 5 % foetal bovine serum (FBS) (BioloT, St. Petersburg, Russia) in the presence of GM-CSF (Sigma- Aldrich, 40 ng/ml) and IFN-alpha (1000 U/ml, Roferon®-А, Hoffmann-La Roche Ltd, Basel, Switzerland,) for 3 days with a subsequent maturation step in the presence of LPS (LPS Е. coli 0114:B4, Sigma-Aldrich, 10 μg/ml) for 48 h.

IL4-DC were generated from an adherent PBMC fraction after incubation in full culture medium in the presence of GM-CSF (40 ng/ml, Sigma-Aldrich), IL-4 (40 ng/ml, Sigma- Aldrich) and 5 % FBS for 5 days followed by an additional maturation step in the presence of LPS for 48 h. In addition to different concentrations of CpG-ODNs, terminal DC maturation was also achieved by incubation with other activators azoximer bromide (AB, NPO Petrovaks Farm, Moscow, Russia) at 2 ng/ml, and double-stranded DNA (dsDNA) at 5 μg/ml.

Intracellular TLR-9 expression in immature DC was assessed in IFN-DC and IL4- DC populations on days 3 and 5, respectively. To this end, cells were permeabilised using a commercially available Fixation/Permeabilization Solution Kit (BD Cytofix/Cytoperm™, San Jose, CA, USA), according to the manufacturer’s instructions. Cells were stained with allophycocyanin (APC)-labelled anti-TLR-9 antibodies (BD PharMingen, San Jose, CA, USA). Matching isotype antibodies labelled with an appropriate fluorochrome were used as negative controls. Percentages of TLR-9-positive DC were calculated based on 10000 events collected during flow cytometric analysis for each sample.

Stimulatory DC activity was assessed in allogeneic mixed leukocyte reactions (allo-MLR) using donor PBMC as responder cells cultivated in round-bottomed 96- well plates (0.1 × 10^6^/well) in RPMI-1640 medium containing 10 % inactivated АВ (group IV) donor serum at 37 °С in a СО_2_-incubator. DC used as stimulator cells were added at a ratio of PBMC : DC = 10 : 1. To assess proliferation, cells were incubated for 4 days, followed by pulse-labelling with 1.0 μCi/well of [3H] thymidine for the last 18 h.

To perform immunophenotyping of IFN-DC, cells were stained with phycoerythrin (PE)-labelled anti-СD14 (Sorbent, Moscow) or anti-OX40L (anti-CD252, BioLegend, San Diego, CA, USA), fluorescein isothiocyanate (FITC)-labelled anti- СD83, anti-CD86 (BD PharMingen) and anti-HLA-DR (Sorbent), and analysed by flow cytometry (FACSCalibur, Becton Dickinson).

Cytokine (TNF-α, IFN-γ) concentrations were measured in 5-day culture supernatants of IFN-DC by ELISA using commercially available kits (Vector-Best, Novosibirsk), according to the manufacturer’s instructions.

Statistical analysis was performed using an analytics software portfolio Statistica 6.0 for Windows (StatSoft, USA). Data is presented as Median (Me) with the interquartile range (IQR, 25–75 % quartiles). Related samples were compared using a nonparametric paired difference test (Wilcoxon signed-rank test), and independent samples were analysed using Mann–Whitney U test; p < 0.05 was considered statistically significant.

## Results

**Fig. 1.**
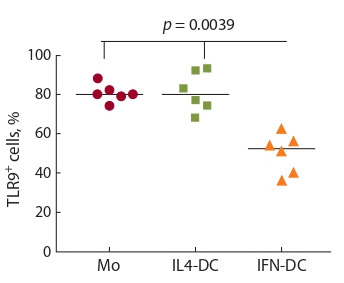
Intracellular TLR-9 expression in monocytes and immature IL4-DC and IFN-DC of healthy donors (n = 6). The data are presented as individual and median values; p – Mann–Whitney U-test.

The assessment of intracellular TLR-9 expression in freshly isolated blood monocytes derived from healthy donors and immature IFN-DC/IL4-DC generated after 3- and 5-days culture, respectively, showed that the proportion of TLR-9-positive cells in monocyte precursors and IL4-DC was at about 80 % level (Fig. 1). In contrast, relative content of TLR-9+ cells in IFN-DC population was significantly lower ranging from 40 to 56 % (Ме 52.5 %, p < 0.05). Nevertheless, the data obtained implies potential sensitivity of DC generated both in the presence of IL-4 and IFN-alpha to the stimulatory effects of CpG-ODN delivering maturation signals.

Therefore, in our next set of experiments we applied dilution series (from 0.5 to 5.0 μg/ml) of CpG-ODN derivatives synthesised here to assess their effect on IFN- DC-mediated ability to stimulate T cell proliferative responses in allo-MLR. Indeed, allostimulatory DC activity is a distinct integral marker of an overall DC activity, being associated with the degree of DC maturation, HLA/co-stimulatory molecule expression, as well as the spectrum and levels of cytokines produced by DC. A “classical” PAMP-activator LPS (at 10 μg/ ml) was used as a positive control, while ODN with μ-modifications, but lacking CpG-dinucleotides (at 1 μg/ml), was used as a negative control. Table 2 shows that LPS treatment caused nearly a 3-fold enhancement of allostimulatory DC activity. All CpG-ODN tested induced IFN- DC maturation, which manifested itself in a statistically significant enhancement of allostimulatory DC activity. In control experiments, ODN lacking CpG motif did not affect functional DC activity in allo-MLR [fold increase (FI) = 1.03; IQR 0.99–1.39].

**Table 2.**
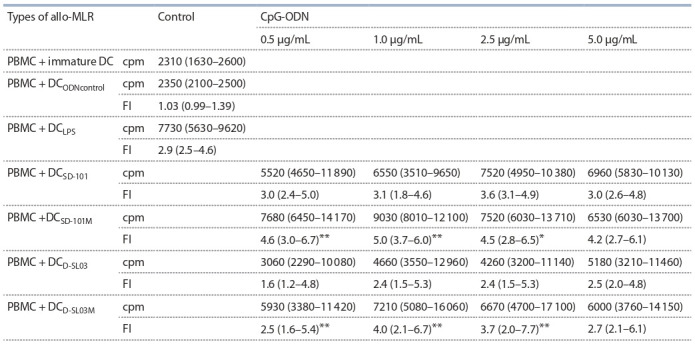
T cell proliferative response in allo-MLR and allostimulatory activity (FI) of IFN-DC generated with CpG-ODNs Notе. The data of two independent experiments (n = 8) are presented as median and interquartile range (in brackets); cpm – count per minute; FI – index of influence. * р < 0.05; ** p < 0.01 – significant difference between μ-CpG-ODN and PS-CpG-ODN (Wilcoxon’s W test).

Characteristically, treatment with CpG-ODN containing mesyl-phosphoramidate (μ-) groups (SD-101М and D-SL03М) at 0.5, 1.0 and 2.5 μg/ml was significantly more effective ( p < 0.01) in inducing terminal IFN-DC maturation, as compared to analogous CpG-ODN that contained thiophosphate groups. Notably, SD-101М exerted the most pronounced effect in these experiments. Thus, SD-101М treatment at the minimal dose tested (0.5 μg/ml) induced clear DC maturation to a degree comparable to that observed in the presence of LPS at 10 μg/ml, while the effectiveness of μ-analogue SD-101 at 1.0 μg/ml was significantly higher than LPS ( p < 0.05).

Next, we performed comparative studies of the effect of μ-analogues CpG-ODN (SD-101M and D-SL03M) and DAMP activators (dsDNA, AB) on allostimulatory activity of two DC subsets (IFN-DC and IL4-DC). Table 3 shows that stimulatory effect of CpG-ODN μ-analogues at 1 μg/ml on functional activity of IFN-DC and IL4-DC in allo-MLR was comparable to that displayed by dsDNA and AB. Furthermore, as compared to IL4-DC, IFN-DC were characterised by higher sensitivity to the chemical compounds studied here, such that all subsequent experiments with CpG-ODN were conducted using IFN-DC cell cultures.

**Table 3.**
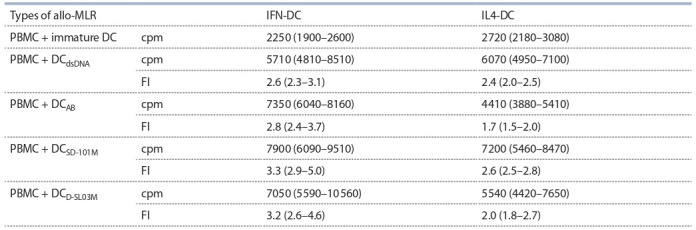
T cell proliferative response in allo-MLR (cpm) and allostimulatory activity (FI) of IFN-DC and IL4-DC generated with different stimuli Notе. The data of two independent experiments (n = 8) are presented as median and interquartile range (in brackets); cpm – count per minute; FI – index of influence. Stimuli: dsDNA 5 μg/ml; AB 2 ng/ml; SD-101М and D-SL03M 1 μg/ml.

**Fig. 2.**
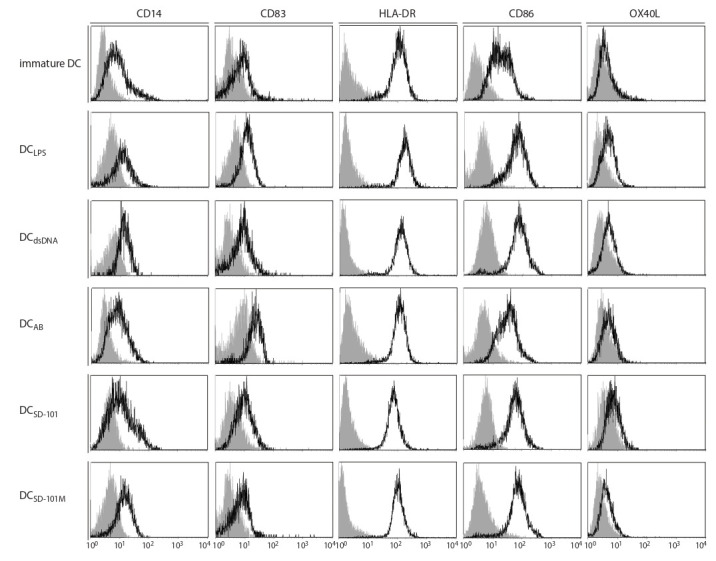
Phenotypic analysis of IFN-DC generated in vitro with different stimuli. Immature IFN-DC were cultured with different stimuli for 24 h followed by flow cytometry analysis of surface molecules CD14, CD83, HLA-DR, CD86, OX40L. Figures show flow cytometry histograms representing the expression of studied markers (bold-line histograms) and matched isotype controls (gray-filled histograms).

To ascertain that the enhancement of allostimulatory DC activity was indeed attributable to terminal DC maturation, we studied immunophenotypic changes occurring in IFN-DC cultivated in the presence of CpG-ODN, as compared to other PAMP and DAMP activators. In this series of experiments, we selected CpG-ODN SD-101 and its μ-analogue SD-101М that showed most pronounced stimulatory activity in previous experiments. Table 4 and Figure 2 indicate that CpG- ODN derivatives studied exerted a clear maturation effect on DC analogous to that observed in the presence of LPS. This maturation effect manifested itself in: (i) reduced percentages of CD14+ monocyte precursors, (ii) increased proportion of mature CD83+DC, and (iii) а clear upward trend for DC expressing a co-stimulatory molecule CD86 ( р = 0.07–0.11). Similar effects were documented in the presence of dsDNA and AB. Interestingly, LPS treatment also significantly enhanced percentages of OX40L-positive DC from 8 to 15 %, which corresponds to 45 % cumulative gain in this particular cell population. The effect of CpG-ODN containing mesylphosphoramidate internucleotide linkages (SD-101М) on OX40L expression was comparable to that observed in the presence of LPS, with percentages of OX40L+DC increasing by 39 % (up to 12.5 %, p = 0.0282). Meanwhile, the effects of dsDNA, AB and SD-101 with thiophosphate internucleotide groups on OX40L expression were less pronounced and did not reach statistically significant levels.

**Table 4.**
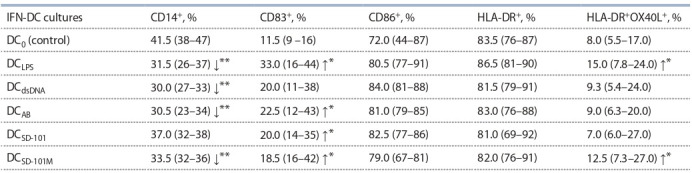
Phenotype of IFN-DCs generated with different stimuli Notе. The data of three independent experiments (n = 7; % of positive IFN-DC) are presented as median and interquartile range (in brackets); Stimuli: LPS 10 μg/ ml; dsDNA 5 μg/ml; AB 2 ng/ml; SD-101 and SD-101М 1 μg/ml. * р < 0.05; ** p < 0.01 – vs control (Wilcoxon’s W test).

In the last experiments we assessed the effects of CpG-ODN SD-101 derivative and its μ-analogue SD-101М on TNF-α and IFN-γ production in 5-day IFN-DC cultures (Table 5) because DC maturation is known to be accompanied by the enhancement of cytokines with pro-inflammatory and Th1-stimulatory activity. As compared to immature cells, LPS-activated DC produced higher levels of TNF-α and IFN-γ ( p < 0.01). CpG- ODN derivatives SD-101 and SD-101М, as well as AB did not affect TNF-α production, while dsDNA treatment increased TNF-α concentrations in DC cultures by 43 %, although this effect was not statistically significant.

**Table 5.**
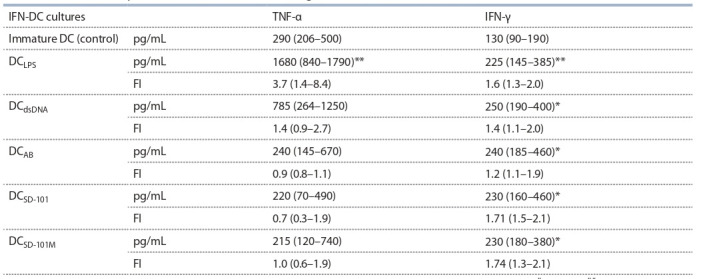
TNF-α and IFN-γ concentration in IFN-DC cultures generated with different stimuli Notе. The data of six independent experiments (n = 13) are presented as median and interquartile range (in brackets); * р < 0.05; ** p < 0.01 – vs control (Wilcoxon’s W test).

Interestingly, SD-101 and SD-101М significantly enhanced the ability of IFN-DC to produce IFN-γ by 71 and 74 %, respectively ( p < 0.05), which was commensurate with LPSmediated effects. Other DAMP activators (dsDNA and AB) also enhanced IFN-γ production by DC.

## Discussion

Data obtained in this study demonstrated intracellular TLR-9 expression in monocyte-derived IFN-DC or IL4-DC. Both DC populations studied here were characterised by sensitivity to CpG-ODN class С derivatives, which enhanced DC-dependent ability to stimulate T cell proliferation in allo- MLR via up-regulation of CD83 differentiation antigen and OX 40L/CD86 co-stimulatory molecule expression, as well as increased IFN-γ production by DC. Interestingly, IFN- DC possessed higher sensitivity to the stimulatory effects of CpG- ODN, as compared to IL4-DC.

TLR-9 expression and CpG-ODN sensitivity have been shown to be characteristic of both plasmacytoid and myeloid DC in murine experimental systems (Behboudi et al., 2000; Iwasaki, Medzhitov, 2004). Early human studies were based on RT-PCR analysis and demonstrated constitutive TLR-9 mRNA expression only in plasmacytoid (but not myeloid or monocyte-derived) DC (Bauer et al., 2001; Krug et al., 2001; Rothenfusser et al., 2002). Nevertheless, in later studies H. Tada et al. discovered TLR-9 mRNA in monocyte-derived DC (mo-DC) and demonstrated enhancement of IL-12p70 and IFN-γ production in mo-DC cell cultures in response to CpG-ODN stimulation (Tada et al., 2005). In line with these observations, V. Hoene et al. identified TLR- 9 protein in mo-DC, which notably was present in the same amounts as in plasmacytoid DC. These authors showed that CpG-ODN class А derivatives stimulated DC maturation and enhanced the ability of DC to stimulate proliferation of allogeneic T cells (Hoene et al., 2006). In this respect, our results constitute another confirmation of human myeloid DC sensitivity to the stimulatory effects exerted by CpG-ODN. In view of the fact that the majority of DC present in tumour microenvironment are of monocyte origin (Veglia, Gabrilovich, 2017), our results imply high clinical potential for CpG-ODN in DC activation protocols, such as those employed in anti-cancer immunotherapy.

Importantly, our study was also first to compare classical thiophosphate CpG-ODN class С derivatives (SD-101 and D-SL03) with their original analogues containing mesyl-phosphoramidate (μ-modified) internucleotide groups (SD-101М and D-SL03М). CpG-ОDN class C derivatives combine immunomodulating properties characteristic of CpG-ОDN class A and B derivatives (Marshall et al., 2005) and also possess pronounced immunostimulatory and anti-cancer effects (Li et al., 2020). For instance, L. Yang et al. demonstrated distinct stimulatory activity of CpG-ОDN D-SL03 derivative, which was able to: (i) activate human B cells, NK cells and T cells in vitro; (ii) intensity the expression of CD80, CD86 and HLA-DR in mononuclear cell cultures, and (iii) furnish anti-cancer effects in a murine model of breast cancer in vivo (Yang et al., 2013). As far as CpG-ОDN SD-101 is concerned, this derivative demonstrated immunostimulatory and anticancer effects during local anti-cancer immunotherapy in patients (Levy et al., 2016; Li et al., 2020). Based on the aforementioned CpG-ODN derivatives, this study was first to synthesise modified analogues SD-101M and D-SL03М with mesyl-phosphoramidate internucleotide groups, which were shown in our previous reports to be more stable to enzymatic cleavage (Miroshnichenko et al., 2019).

A comparison analysis of CpG-ODN derivatives performed here showed that μ-modified analogues were superior in enhancing allostimulatory DC activity, as compared to CpG-ODN containing thiophosphate internucleotide groups. Moreover, it was the particular μ-type SD-101 derivative (SD-101М) that was found to enhance OX40L [an important co-stimulatory molecule regulating the intensity of T cell proliferation in allo-MLR (Ukyo et al., 2003)] expression in IFN-DC population, i. e. displayed properties characteristic of LPS.

It should be stressed that TLR-4-specific ligand LPS constitutes a powerful DC maturation activator, which is widely used in various in vitro settings as a positive control. TLR-4- mediated signalling is known to increase a downstream augmentation of co-stimulatory molecule expression, pro-inflammatory cytokine production, DC-mediated stimulatory activity with respect to allogeneic T cell proliferation, and Th1-dependent responses (Cehim, Chies, 2019), thus supporting full-fledged immunological functionality of activated DC. Interestingly, V. Hoene et al. showed that stimulatory effect of D19 (CpG-ODN class А compound) on maturation and allogeneic activity of DC was lower than LPS (Hoene et al., 2006). Meanwhile, this study showed that SD-101М activity was in fact higher than LPS, with SD-101, D-SL03 and D-SL03М-associated activity being comparable to that of LPS. Enhancement of IFN-γ production in response to CpG-ODN (SD-101 and SD-101М) treatments was also commensurate to LPS.

It should be mentioned that we compared CpG-ODN class C derivatives not only with LPS, but also with such DAMP activators as human dsDNA and azoximer bromide. Stimulatory effects of SD-101М on DC maturation and allostimulatory activity was found to be commensurate with dsDNA and AB, which complements our previous studies that showed stimulatory effects of dsDNA on DC maturation and allostimulatory activity (Alyamkina et al., 2010; Orishchenko et al., 2013). However, this study also described for the first time an ability of an AB-based polymeric adjuvant developed in Russia to stimulate in vitro maturation of DC derived from monocytes in the presence of IL-4 or IFN-α, as well as to enhance DC allostimulatory activity. These findings provide an important experimental support for the effective clinical application of this adjuvant in anti-viral vaccine formulations. Of note, SD- 101М outcompeted for a number of parameters (for example, induction of OX40L expression and IFN-γ production) dsDNA and AB.

## Conclusion

Taken together, data obtained in this study demonstrated pronounced stimulatory effects of CpG-ODN class С derivatives (SD-101 and D-SL03) on human myeloid DC, which were commensurable to that displayed by PAMP (LPS) and DAMP (dsDNA and AB) activators, while the effects of a mesyl-phosphoramidate (μ-) analogue SD-101М were even stronger. Further studies in murine experimental models are needed to analyze the efficacy of the μ-modified CpG-ODN (SD-101M with mesyl-phosphoramidate internucleotide groups) in anti-cancer immunotherapy.

## Conflict of interest

The authors declare no conflict of interest.
